# Editorial: Health effects of natural compounds from plants

**DOI:** 10.3389/fnut.2026.2002556

**Published:** 2026-10-09

**Authors:** Jing Gao, Hui Chen, Mengjie Li, Wenlong Wang

**Affiliations:** 1 Research Institute of Oil Tea Camellia, Hunan Academy of Forestry, State Key Laboratory of Woody Oil Resources Utilization, National Engineering Research Center for Oil Tea Camellia, Changsha, China; 2 Yuelushan Laboratory, Changsha, China; 3 Texas Tech University Lubbock, Lubbock, TX, United States; 4 Sun Yat-sen University, Guangzhou, China; 5 Nutrition and Physical Exercise Health Promotion Workstation, Faculty of Physical Education and College of Medicine, Pingdingshan University, Pingdingshan, China

**Keywords:** epigenetic regulation, functional foods, gut microbiota, molecular nutrition, multi-omics, oxidative stress, plant-derived natural compounds

## Introduction

Plant-derived natural compounds (PNCs), including flavonoids, alkaloids, polysaccharides, and essential oils, are rapidly transitioning from traditional ethnopharmacology into mainstream molecular nutrition. Their structural diversity and multi-target profiles make them ideal candidates for mitigating oxidative stress, chronic inflammation, and metabolic dysfunctions. This Research Topic, “*Health effects of natural compounds from plants*,” curates 33 peer-reviewed articles, comprising original research papers and comprehensive reviews (Tian et al.; Alomari et al.; Nie et al.; Li Y. et al.; Long et al.; Hong et al.; Lei et al.; Wang et al.; Chen Y. et al.; Lin et al.; Lu et al.; Zeng et al.; Park et al.; Guo et al.; Liu et al.; Song et al.; Yin et al.; Yuan et al.; Mo L. et al.; Bin et al.; Chen H. et al.; Lian et al.; Mo K. et al.; Meng et al.; Ma et al.; Nong et al.; El-Saadony, Saad, Fahmy et al.; Li S. et al.; Saad et al.; El-Saadony, Saad, Mohammed et al.; Javed et al.; Abid et al.; Li M. et al.), detailing the therapeutic applications, delivery systems, and underlying mechanisms of these bioactives. Mechanistic depth, rather than phenotypic description, unifies this Research Topic. Instead of merely reporting phenotypic changes, these studies investigate specific signaling cascades, epigenetic modifications, and microbiota-mediated biotransformations.

## Advancing mechanistic insights in oncology and metabolism

To elucidate the anti-tumor properties of these products, researchers are increasingly relying on advanced biological models. Using 3D colorectal cancer organoids, Tian et al. evaluated berberine and demonstrated that this alkaloid actively restrains tumor expansion by blocking cell cycle progression and suppressing lipid synthesis. Combination therapies offer another pragmatic route to clinical translation, as illustrated by Alomari et al. through pairing metformin with parthenolide in a murine breast cancer model to inhibit tumor growth through apoptosis induction and angiogenesis blockade. Such drug repurposing maximizes metformin's established safety profile while utilizing parthenolide's pro-apoptotic benefits. Beyond direct cytotoxicity, the Notch signaling pathway has emerged as a promising target for bioactive compounds in cancer intervention, which was systematically reviewed by Nie et al. Further exploring the intersection of gut microbiota and immunotherapy, Li Y. et al. positioned Polygonatum polysaccharides as modulators that enhance autophagy-dependent PD-L1 clearance. In parallel, Long et al. examined the cGAS-STING pathway as a central innate immune sensor, highlighting its potential as a therapeutic target for natural plant products in digestive disorders. Bibliometric analysis by Hong et al. mapping research on curcumin and immunity over the past two decades revealed a clear shift from descriptive observations toward molecular mechanisms and clinical applications.

While oncological mechanisms form a major focus, this Research Topic equally highlights the metabolic regulatory potential of plant natural products. Dietary intervention using oligosaccharides extracted from *Bletilla striata* successfully alleviates high-fat diet-induced metabolic-associated fatty liver disease (MAFLD) in mice by remodeling the gut microbiota and host metabolism (Lei et al.). Similarly, *Artemisia argyi* essential oil modulates lipid metabolism via linoleic acid and glycerophospholipid pathways in obese mice (Wang et al.). Renal interstitial fibrosis is attenuated by isorhamnetin through targeting TWEAK/Fn14-mediated epithelial-mesenchymal transition (Chen Y. et al.). In diabetic zebrafish, *Polygonatum sibiricum* polysaccharides enhance pancreatic β-cell function by mitigating mitochondrial oxidative damage via the AMPK-SIRT1 pathway (Lin et al.). Polysaccharides isolated from *Berberis dasystachya* M. fruits protect RINm5F pancreatic β-cells against H_2_O_2_-induced oxidative damage (Lu et al.). Furthermore, Zeng et al. reviewed the clinical benefits of food-medicine homologous herbs for patients with diabetic kidney disease, while Park et al. reported that black soybean and black rice extracts ameliorate aging-induced muscular decline. Adding to these metabolic insights, catechin inhibits oxidized low-density lipoprotein-induced ferroptosis in vascular smooth muscle cells, thereby alleviating and stabilizing atherosclerosis (Guo et al.). Finally, Liu et al. analyzed the synergy between cyanidin-3-O-glucoside and catechin in absorption, transport, and lipid metabolism.

## Gut barrier integrity and epigenetic immune regulation

As the primary interface for nutritional immunology, the gastrointestinal tract is a prime target for PNC intervention. Applying a comprehensive multi-omics approach, Song et al. uncovered an intricate network of mucosal repair pathways through which *Panax notoginseng* extract alleviates ulcerative colitis. PI3K-AKT signaling serves as the core mechanism by which *Mosla chinensis* essential oil fortifies the intestinal barrier against colitis (Yin et al.). Luteolin also attenuates lipopolysaccharide-induced damage in IPEC-J2 cells by enhancing mitophagy via AMPK signaling pathway activation (Yuan et al.), whereas dietary fiber from burdock root ameliorates functional constipation in aging rats by regulating intestinal motility (Mo L. et al.).

A conceptual advance in this Research Topic comes from the epigenetic regulation of immune responses by plant small molecules. Addressing how small plant molecules operate at the genomic level, Bin et al. revealed that antroquinonol suppresses IL-1β secretion in macrophages by driving DNA methylation at the Tlr4 promoter rather than simply blocking a receptor. This challenges the view of natural compounds as mere enzyme inhibitors, indicating that dietary phytochemicals can reshape immune responses through durable chromatin modifications. The polypharmacology of plant products, once considered a hurdle in targeted drug development, is now emerging as a strategic advantage for managing multifactorial diseases.

## Tissue repair, radiation protection, and advanced delivery systems

Beyond metabolic and inflammatory control, the applications of PNCs extend to complex tissue repair. Jujube pectic oligosaccharides show strong reparative effects on bone marrow hematopoietic failure, providing empirical support for the emerging gut-bone marrow axis (Chen H. et al.). For patients undergoing radiotherapy, collateral tissue damage remains a severe challenge; berberine preserves and significantly augments salivary gland secretory function post-radiation (Lian et al.). Furthermore, Mo K. et al. demonstrated that shikimic acid attenuates oxidative stress-induced senescence in 3D4/21 cells by modulating PARP1 activity and DNA damage response. To further accelerate the discovery of such regenerative agents, Meng et al. validated the zebrafish model as an efficient genetic screening platform.

Translating these *in vitro* successes into *in vivo* efficacy demands effective delivery systems. Ma et al. discussed how egg-based carriers can effectively encapsulate omega-3 fatty acids and antioxidants, bypassing classic bioavailability bottlenecks.

## Translational applications: functional foods and comprehensive reviews

Translating basic research into practical applications is a core mission of nutritional science. Upcycling plant side-streams offers a highly sustainable trajectory for agricultural valorization, as demonstrated by Nong et al., who found that supplementing broiler diets with fermented passion fruit (*Passiflora edulis* Sims) peel significantly improves growth performance, intestinal morphology, and cecal microbiota. El-Saadony, Saad, Fahmy et al. provided a foundational overview of flavonoids, detailing their immunological properties and broad food industry applications. Recent advances in *Tremella fuciformis* polysaccharides—covering isolation, structures, bioactivities, and applications—were reviewed by Li S. et al.; Saad et al. comprehensively analyzed dietary polyphenols and human health, focusing on sources, biological activities, and bioavailability. Curcumin was extensively reviewed by El-Saadony, Saad, Mohammed et al., covering its biological activities, immunological properties, and bioavailability challenges. Javed et al. highlighted black rice as an emerging functional food with bioactive compounds and therapeutic potential, while Abid et al. updated the phytochemical profile and therapeutic potential of Aloe vera. Additionally, Li M. et al. reviewed omega-3 fatty acids, focusing on multi-target mechanisms and therapeutic applications in neurodevelopmental disorders and epilepsy.

## Future perspectives

This Research Topic demonstrates that research on plant-derived natural compounds has moved beyond descriptive phenomenology into multi-omics and epigenetic investigation. These 33 contributions enrich our understanding of the nutrition-immunology nexus and provide solid scientific support for next-generation functional foods, targeted drug delivery systems, and integrative clinical therapies.

Several challenges remain. Bridging preclinical mechanisms and human efficacy will require well-designed clinical trials. Low bioavailability and compound instability continue to limit *in vivo* efficacy, calling for deeper investment in microencapsulation and nanoencapsulation technologies. Standardizing plant material sourcing and extraction protocols is equally important for ensuring cross-study reproducibility. As multi-omics profiling becomes routine, systems biology will further enable precision nutrition. Finally, underutilized plant resources and agricultural waste streams represent untapped reservoirs for public health and sustainable bioeconomy development.

We thank all contributing authors and reviewers for their rigorous work. Their work lays the groundwork for translating phytochemical mechanisms into functional foods and clinical therapies.

